# Editorial: Exploring metabolomic diversity in plant species by NMR-based and mass-based spectrometry

**DOI:** 10.3389/fpls.2023.1248781

**Published:** 2023-07-18

**Authors:** Gabriella Saviano, Debora Paris, Maria Iorizzi

**Affiliations:** ^1^ Department of Biosciences and Territory, University of Molise, Pesche, Italy; ^2^ Institute of Biomolecular Chemistry, National Research Council (CNR), Pozzuoli, Italy

**Keywords:** plant secondary metabolites, metabolomics, multivariate data analysis, natural products, technological advances, GC-MS, LC-MS, NMR

Metabolomics has been defined as the analysis of all metabolites in an organism through the simultaneous detection of all-natural chemical components in a given biological system. With the development of metabolic profiling technologies suitable for large-scale measurements, metabolomics is now playing a significant role in basic plant biology, with its mechanisms, and in applied biotechnology. Plants produce a huge amount of chemicals that play an essential role in the interaction of plants with its natural environment and their ability to adapt. Metabolomics is, therefore, a powerful tool in plant ecology and biodiversity research. Furthermore, natural products of plant origin have long been considered a valuable source of lead compounds for drug development. Two main analytical techniques, MS and NMR are the current strategies to explore highly complex and diverse plant metabolomes. These methodologies have played a dominant role since the early days of metabolomics, and their strengths and limitations have been recognized. In this context, we launched our Research Topic by inviting researchers to contribute in the exploring metabolomic diversity in plant species, using spectroscopic and spectrometric data. Indeed, current metabolomics strategies are mainly based on the main approaches: nuclear magnetic resonance (NMR) spectroscopy, gas chromatography-mass spectrometry (GC-MS) and liquid chromatography-mass spectrometry (LC-MS). Furthermore, the increasing development of statistical methods based on the analytical data, allowing for the finely extraction of information from the huge data provided by analytical technologies thus amplifying the possibility of differentiating species and highlighting specific compounds even in limited quantities.

This Research Topic brings together four original research articles (Nie et al., Blommaert et al., Beatrice et al., Wang et al.) and one review (Kumar et al.). The different contributions collected here, highlight diverse analytical methodologies supported by different processing of analytical data. This wide versatility and application of the techniques used, allowed for more efficient management of metabolomic data.

More specifically, Nie et al. report for the first time desorption electrospray ionization (DESI) in combination with Q-TOF/MS to reveal the spatial distribution of metabolites in the cross-section of Isatidis Radix and distinguish samples with different quality characters based on the ion images (MSI) and pattern recognition method (OPLS-DA). After all, markers related to the quality characters of Isatidis Radix were discovered, which exerted stronger spatial signals in good-quality samples and showed significant influence on differentiation.

Of a completely different nature was the contribution of the work of Blommaert et al. The authors elucidated the transfer of Cd from soil to the nib (seed) in a high Cd accumulating cacao cultivar through Cd stable isotope fractionation, speciation (X-Ray Absorption Spectroscopy), and localization (Laser Ablation Inductively Coupled Plasma Mass Spectrometry). In this context, mass spectrometry has been used indirectly as a metal detection and analysis technique. In fact, LA-ICP-MS is widely used as a powerful analytical technique for solid sample analysis in a variety of scientific fields including biological and environmental sciences.

The research of Beatrice et al. concerns the use of plants and metabolic profiles in the development of new technologies and their potential applications. This work analyses the differentiation of morphological and metabolic profiles of plants in response to physiological or pathological conditions. The morphological characteristics of two common species of aromatic plants, *Ocimum basilicum* and *Mentha x piperita*, were analysed to understand if the CoeLux^®^ type of light can allow their growth and development in indoor environments. In addition, through the application of GC-MS analysis, the composition of essential oils (EO) was studied as a possible indicator of the activation of different metabolic pathways. In this study, it was hypothesized that the growth of aromatic plants *M. piperita* and *O. basilicum*, under CoeLux^®^ lighting systems is a feasible strategy to improve biophilic approaches in indoor environments that include both plants and artificial sunlight.

Wang et al., on the contrary, employed the multi-informative molecular networking (MIMN) approach to construct the anti-inflammatory metabolomic pattern of a heat-clearing herb, *Scophularia ningpoensis* Hemsl, from data obtained by tandem MS spectrometry. The results indicated that a major cinnamic acid glycoside, angoroside C, was responsible for the heat-clearing effect of *S. ningpoensis* and should be selected as the chemical marker.

To close, the review of Kumar et al. emphasizes the importance of nuclear magnetic resonance (NMR) among existing methods for measuring sap flow in plants as non-invasive approach. Indeed, several advances have recently been made that have enabled the production of portable NMR instruments for measuring sap flow in plants. The development of a noninvasive, portable and inexpensive instrument that can be easily used under field conditions would greatly improve the ability to measure vegetation responses to environmental changes.

In conclusion, the progressive development of advanced MS and NMR technologies and the ability to interface with increasingly comprehensive databases enables the accurate identification and quantification of many metabolites in plants. Nevertheless, compared with the acquisition of data, the subsequent processing and mining of data are the bottleneck problems facing metabolomics. Although mass spectrometry has the traits of high resolution, good precision, and maximum throughput (Guo et al., 2023), and NMR is capable of detecting metabolites present in solution at concentrations above 1mM, with little or no prior knowledge (Mulder et al., 2023), the research progress of metabolomics is still slow due to the complexity of organisms, the huge number of metabolites, and unrepeatable experimental protocols in different laboratories. Despite this, several researches think that the two techniques are very complementary, and the weaknesses of one are compensated by the strengths of the other.

## Author contributions

All listed authors contributed directly and intellectually to the work and approved its publication.
